# A Case of Red Retching?

**DOI:** 10.1097/PG9.0000000000000230

**Published:** 2022-07-25

**Authors:** Sabrina A. Karim, Tao Tao Holmes, Allison J. Wu, Daniel Kamin

**Affiliations:** Department of Pediatrics, Boston Children’s Hospital, Boston, MA; Department of Pediatrics, Harvard Medical School, Boston, MA; Department of Pediatrics, Boston Medical Center, Boston, MA; Department of Pediatrics, Boston University, Boston, MA; Division of Pediatric Gastroenterology, Hepatology and Nutrition, Boston Children’s Hospital, Boston, MA

**Keywords:** adolescent, diagnostic, emergency, emesis

## Abstract

This piece features a 14-year-old young man who presented with epigastric pain and bright red emesis. His father brought both a photo and sample of the vomitus, which guided initial management in one direction, and then on closer inspection, diverted his diagnostic trajectory. Through a traditional case report and accompanied image and prose, we explore how we process and reinterpret visual data to help guide our management of hematemesis.

## A CASE OF RED RETCHING

A 14-year-old previously healthy male presented with 1 day of moderate epigastric pain and 3 episodes of bright red emesis. He reported 1 month of daily nonbilious, nonbloody emesis upon waking and postprandially. Associated symptoms included nausea, weight loss, poor appetite, and early satiety. The patient reported no changes to his stool pattern and no bright red or dark tarry stools. Family history was negative for clotting or bleeding disorders.

On presentation to an outside emergency department, the patient was afebrile and his vital signs were normal, without hypotension, or tachycardia. On exam, the patient had normal perfusion and mild epigastric tenderness on palpation. His blood work showed no anemia, with normal chemistries, liver tests, coagulation studies, and lipase. The vomitus was reportedly gastroccult positive. The patient was transferred to our institution for admission for further work-up and observation for upper gastrointestinal bleeding. Based on the urgent transfer and history provided, the patient was scheduled for an endoscopy the next morning.

During inpatient rounds the next day, the patient’s father provided photographs of the patient’s emesis, which immediately stood out to be unusual in appearance and atypical for hematemesis. Specifically, the discrete, uniform and cherry red contents separated by clear, foamy saliva were not consistent with hematemesis. Upon further history taking, the patient endorsed consuming a large quantity of red gummy candies the night prior. In private, he also endorsed frequent cannabis usage and relief of his nausea and vomiting after taking hot showers. He was diagnosed with cannabinoid hyperemesis syndrome and likely associated dysmotility (1). His clinical appearance, blood work, and spontaneous resolution of symptoms were reassuring. He was counseled on cannabis cessation and discharged with omeprazole and capsaicin.

This case of “red retching” is a reminder that all that resembles blood may not truly be blood, and in cases of vomiting, red food dyes and coloring can be duplicitous. This is also the case for hematochezia and melena, where cases of “red dye-arrhea” (2) and culprits such as cake frosting, Kool-Aid, and cefdinir (3) remind us of the value of deep investigation, both visually and on history taking. For our patient, through further confidential questioning, we were able to uncover his frequent cannabis use leading to cannabinoid hyperemesis syndrome, an emerging challenge in pediatrics (4). Our patient was spared his scheduled endoscopy because the hematemesis was reinterpreted as gummy candy “red retching” through careful inspection of a critical photograph.

For a unique illustration of this case, please see accompanying poem (Supplemental Digital Content 1, http://links.lww.com/PG9/A90).

**FIGURE.**
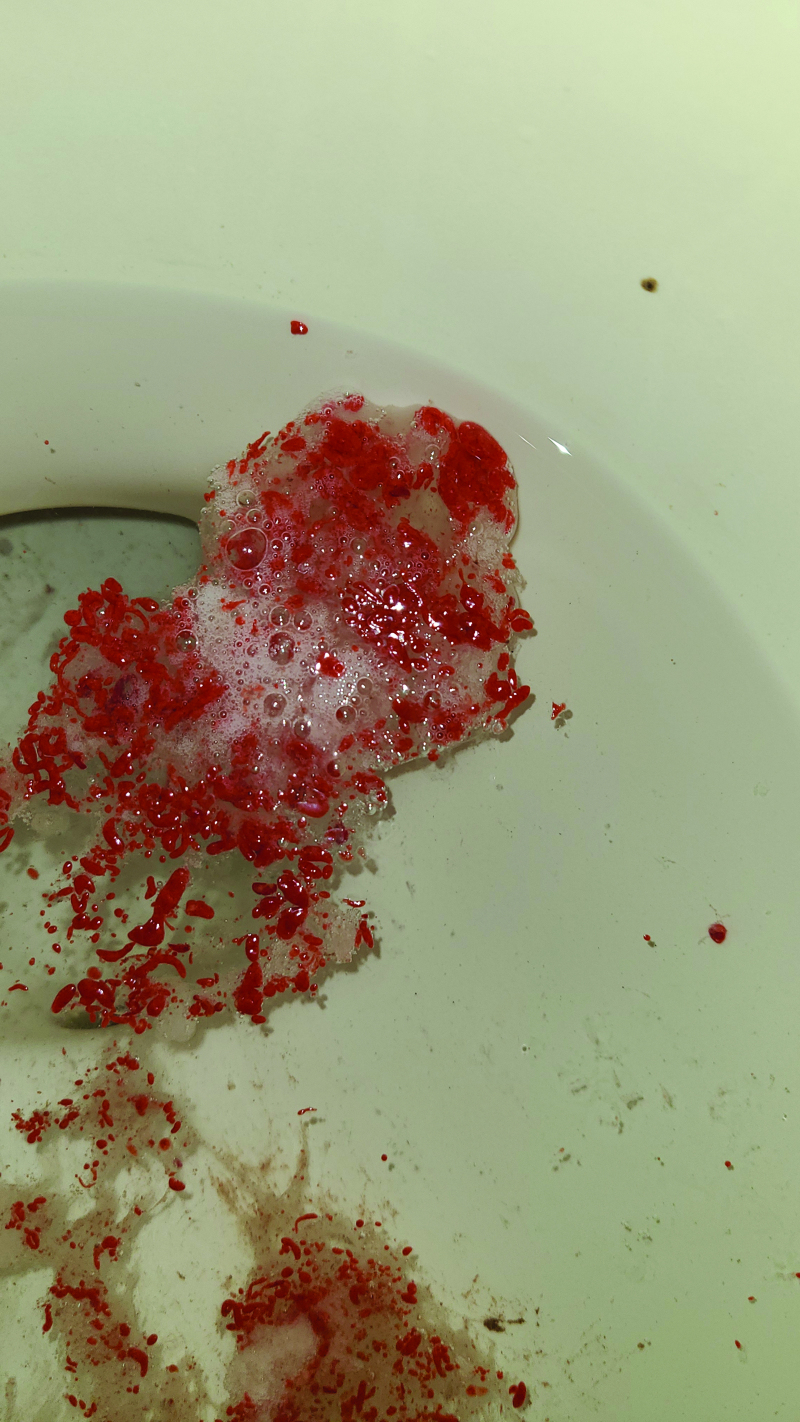
Image of the sample of vomitus.

## ACKNOWLEDGMENTS

Informed patient and guardian consent was obtained for publication of case details.

## Supplementary Material


