# Supplementary material for: A Case of Red Retching?
Source: JPGN Rep. 2022 Jul 25;3(3):e230. doi: 10.1097/PG9.0000000000000230 (PMC10158285; doi:10.1097/PG9.0000000000000230)
Supplement: Supplementary file 1 [file pg9-3-e230-s001.pdf]

Supplemental Digital Content:

A Case of Red Retching?

Vomitus red, what to do? Here is a clinical conundrum for you!

One day of moderate epigastric pain
In terms of the symptoms, this one was the main
Although there was in fact an additional plight
Bright red emesis, its color was bright

‘Twas a healthy male, his age but fourteen
Such a concern he had never before seen
Bowel movements were non-bloody and soft in form,
Vitals were normal, perfusion within norm
As were other labs, including complete blood count
Within an expected amount
No tachycardia nor hypotension were shown
Per the family history, no bleeding disorders were known

A photo of the emesis from earlier in the day
Was provided by father, with a sample (hooray!)
The sample was found to be gastroccult plus
Positive, that is, and we proceeded thus:

Admission for work-up of upper GI bleed
Differential diagnoses we were careful to heed
(Included were the syndrome of Mallory-Weiss,
malrotation, and gastritis—though none would be nice)

The next morning arrived, and during patient rounds,
Something surprising the team suddenly found!
In close review of the photo, the vomitus did not appear bloody
Rendering the situation a little less muddy

In fact, the vomitus was appearing more like…
Gummy candy, perhaps? À la Mike & Ike?
As it turns out, the fateful night prior
Copious gummy bonbons (perhaps a bag entire?!)
Had been gobbled by our patient with delight
And now, ‘twas our doctorly duty to make things right

We performed an upper gastrointestinal series
To rule out any possible malrotation queries

A diagnosis of gastroparesis, we did then conclude
This case of “red retching” was all tied to food!

No upper endoscopy was ultimately needed
And our patient, heading home, has definitely heeded
A warning that eating too many red gummies
Can lead to confusion and very unhappy tummies
